# The Evaluation of Antibacterial, Antifungal and Antioxidant Activity of Methanolic Extract of *Mindium Laevigatum* (Vent.) Rech. F., From Central Part of Iran

**Published:** 2013-02-13

**Authors:** Masoud Modaressi, Roia Shahsavari, Farhad Ahmadi, Mehdi Rahimi-Nasrabadi, Ramin Abiri, Ali Mikaeli, Hossein Batoli

**Affiliations:** 1 Department of Pharmacognosy, Faculty of Pharmacy, Kermanshah University of Medical Sciences, Kermanshah, IR Iran; 2 Department of Medicinal Chemistry, Faculty of Pharmacy, Kermanshah University of Medical Sciences, Kermanshah, IR Iran; 3 Department of Analytical Chemistry, Faculty of Science, Imam Hossein University, Tehran, IR Iran; 4 Department of Microbiology, Faculty of Medicine, Kermanshah University of Medical Sciences, Kermanshah, IR Iran; 5 Isfahan Research Center of Natural Sources and Agriculture, Kashan Station, Kashan, IR Iran

**Keywords:** Methanolic Extract, Antioxidant, Antifungal, Antibacterial

## Abstract

**Background:**

*Mindium laevigatum* (Vent.) Rech. F. plant grows in central part of Iran. And used by local people as medical plant.

**Objectives:**

The purpose of this study was to investigate the in vitro antibacterial, antifungal and antioxidant activities of the methanolic extracts of aerial and flower parts of plant.

**Materials and Methods:**

The leaves and stem and flower of bark from *M. laevigatum* were separately collected, air-dried and powdered. Then the plant species extracts were prepared with methanol, water 80:20 and two polar and non-polar subfractions were realized. The antioxidant activity was evaluated by scavenging the radicals 1, 1-diphenyl-2-picrylhydrazyl radical (DPPH), β-Carotene linoleic acid assay and reducing power methods. The antifungal and antibacterial evaluation was performed by disc diffusion and minimum inhibitory concentration methods.

**Results:**

The total phenolic analysis of subfractions found 182 ± 4.2 µg.gr^-1^ for polar and 158 ± 3.9 µg.gr^-1^ for non-polar extracts. The antifungal activity of the extracts against the various fungal varied from 14.0 to 34 mm. MIC values from 50 to 400 µg.mL^-1^ were satisfactory when compared with other plant products. The antibacterial results revealed that the subfraction extracts are mostly effective against *Staphylococcus aureus*. The antioxidant results showed polar subfraction has more activity against non-polar subfraction.

**Conclusion:**

These findings demonstrated that the extract of *Mindium laevigatum* has remarkable in vitro antifungal and antioxidant activity.

## 1. Background

Currently, great progress has been made in the use of natural compounds, plant materials and herbal medicine for improving healthy dietary intake and the quality of life (1-6). In the medicinal field, plant materials play a major role in primary health care as therapeutic remedies in many developing countries (7, 8). In the food industry, the biological proprieties of plant extracts have been investigated for the possible use of essential oils and/or solvent extracts of plants for the control of pathogenic microorganisms or as antioxidants (9-11). The request for reduced use of the synthetic antioxidants such as butylated hydroxyl toluene (BHT) and butylated hydroxyl anisole (BHA) in the food industry has triggered the need for the development of alternative active compounds, which are harmless to the consumers and to the environment while being useful for the protection of foods from microorganisms and free radicals (12, 13).

Consequently, the reports of antioxidant, antimicrobial and anti-fungal properties of local plants are of great interest and importance (14-16). *Mindium laevigatum* (Vent.) Rech. F. was introduced as a member of Campanulaceae family in the central part of Iran by Moosavi and coworkers (IRAN 10909F), (17). Some of the species belonging to Campanulaceae family have been previously investigated from different points of view: in vitro amoebicidal activity of *Origanum laevigatum* on *Acanthamoeba castellanii* cysts and trophozoites (18), cytotoxic and anti-inflammatory cembranoids of *Lobophytum laevigatum* (19). Recently, Masoum et al. reported the volatile chemical constituents of *Mindium laevigatum* by GC-Mass (20). In 2012, the Güvenç et al. studied the anti-inflammatory, wound healing and antioxidant activities of the several Campanulaceae species native of Turkey (21). According to our knowledge, no report exists regarding the antifungal, antibacterial and antioxidant activities of the solvent extract of *Mindium laevigatum* (Vent.) Rech. F. This species is a dense bush with a characteristic aroma and small green leaves that is distributed in central parts of Iran and commonly known as ‘‘Gole Shekafteh” by the local people. This study is divided into two main objectives as an investigation of antifungal and anti-microbial activity of the polar and non-polar sub-fractions of methanolic extracts of *Mindium laevigatum* (Vent.) Rech. F. by disc diffusion and MIC against some pathogenic fungi and bacterial, and the antioxidant activity evaluation of extracts by three separate methods, namely inhibition of free radical 2, 2-diphenyl-1-picrylhydrazyl (DPPH), β-carotene–linoleic acid system and reducing power.

## 2. Objectives

The useful findings of the present study for the protection of foods against contamination especially *S. aureus* was also discussed.

## 3. Materials and Methods

### 3.1. Plant Material

*Mindium laevigatum* (Vent.) Rech. F., plant was collected from nearby Kashan (Isfahan province, Iran). The taxonomic identification of plant material was confirmed by a senior plant taxonomist, H. Batoli, in Isfahan Research Center of Natural Sources and Agriculture, Iran. The plant was dried in the shade, and ground in a grinder. The voucher specimen has been deposited at the Herbarium of the Isfahan Research Center of Natural Sources and Agriculture, Kashan Station, Kashan (IRAN 10909F).

### 3.2. Preparation of Plant Extracts

The extraction of an air-dried and finely ground sample was carried out using the method developed by Atmani (22) with minor modifications. Plant powder (20 g) was macerated and stirred in MeOH: H_2_O (80:20 v/v%, 200 mL) during 6 hours at room temperature. The residue was re-extracted until extraction solvents became colorless. The obtained extracts were filtered over Whatman No. 1 paper and the filtrate was collected, and then the solvent was removed by a rotary evaporator at 40 °C to obtain dry extract. The dried yield was partitioned into chloroform and water. The resulting phases were dried giving rise to organic and aqueous extracts. At the end, these two subfractions were tested for various activities.

### 3.3. Antibacterial and Antifungal Activity

#### 3.3.1. Bacterial Strains and Fungi

The bacterial strains used in the experiment were kindly provided by Dr. R. Abiri from the Microbiology Laboratory of Kermanshah University of Medical Sciences, Kermanshah, Iran. The antimicrobial activities of polar and non-polar subfractions of the methanolic extract of plant were individually tested against a panel of bacterial strains including: three Gram-positive bacteria such as: *Enterococcus feacalis* (ATCC 29122), *Staphylococcus aureus* (ATCC 12600), *Staphylococcus epidermidis* (A233) and four Gram-negative bacteria such as *Pseudomonas aeruginosa* (ATCC 27853), *Morganella*, *Citrobacter freundii* and *E. coli* (ATCC 11522).

The pathogenic fungi including *Aspergillus candidus, Aspergillus niger* (ATCC 6275) and *Candida albicans* (ATCC 64550) were provided from the Laboratory of Fungal, Kermanshah University of Medical Sciences, Kermanshah, Iran.

#### 3.3.2. Determination of Minimum Inhibitory Concentration (MIC) for Bacterial Strains

The minimal inhibitory concentration (MIC) values were also studied for the polar and non-polar extracts of plant similar to our earlier study (14). The extracts were first dissolved in H_2_O: DMSO (95:5 v/v%) and diluted to give rise a concentration of 800 μg mL^-1^. For making a series of solutions with concentration range of 12.5 to 800 μg mL^-1^ the serial dilution method was used. The target microorganisms were first cultured in Mueller-Hinton broth (MHB) for 24 hours in day and night at 37 °C, and then diluted with 0.5 McFarland standard turbidity. The suspensions were diluted again with a ratio of 1/1000 ratio by Mueller-Hinton broth. One hundred µl of Mueller-Hinton broth containing diluted bacteria (1.5×108 CFU mL^-1^) and 100 µl of aliquot from the solutions of the polar and non-polar subfractions were added into 96-well microtitres plates. A positive control (containing inoculums but no extracts) and negative control (containing extracts but no inoculums) were included on each microplate. The contents of the wells were mixed and the microplates incubated at 37 ºC for 24 hours under microaerophilic conditions. The MIC was defined as the lowest concentration of the compounds to inhibit the growth of microorganisms and measured visually.

#### 3.3.3. Antifungal Activity Assays

Antifungal activity of extracts was studied by both disk diffusion and MIC methods (23) with some modifications. Firstly a sterile potato dextrose agar (PDA) medium (Merck) was prepared and transferred into Petri plates of 90 mm diameter. The diameter of the sterile disc was 10 mm (Whatman No. 1). Two solutions of the polar and non-polar extracts (800 μg mL^-1^ in H_2_O: DMSO 95:5 v/v%) were made. In order to have a uniform fungal growth on both control and test plates, the spore suspension was streaked over the surface of the PDA by using a sterile cotton swab. After that, the discs were inoculated on the agar plates and 10 µl from each of the extract was put on the discs. 10 microliter of dilution solvent (H_2_O: DMSO 95:5v/v%) and 10 µl of ketoconazole (100 μg mL^-1^) were added to the discs as negative and positive controls, respectively. The plates were then incubated at 30 °C for 24 hours in order to get a reliable fungal growth. Diameters of microbial inhibition zones were measured using vernier calipers. The experiment was repeated for three times in three days and diameter of the inhibition zone was determined by averaging the radial inhibition zone of three replicates. Minimum inhibitory concentrations (MICs) determination was carried out by a serial dilution method in 96-well microtiter plates with potato dextrose agar (PDA) as the growth medium (24). Several solutions with different concentrations (12.5-800 μg mL^-1^) were prepared. 100 µl of the potato dextrose agar containing 1.0/×106 cells/mL (24), and 100 µl of a test solution of a specific concentration were added to each well. 100 µl of ketoconazole (100 μg mL^-1^) and 100 µl of PDA were used as positive and negative control, respectively. The micro plates were incubated for 24 hours at 30 °C in over day and night. After 24 hours incubation at 30 °C, fungi growth was evaluated visually and the minimum inhibitory concentration (MIC) was determined. The MIC was defined as lowest concentration that completely inhibited fungal growth (MICs).

### 3.4. Antioxidant Studies

#### 3.4.1. The Free Radical Scavenging of DPPH Radicals

The free radical scavenging activities of polar and non-polar extracts of the plant were measured by using 1,1'-diphenyl-2-picryl-hydrazyl (DPPH) (25) with some modifications. 2.0 mL of various concentrations of the polar and non-polar extracts were added to 1.0 mL of methanolic solution of DPPH (5 mM). The mixture was vigorously shaken and stood at room temperature for 60 minutes in a dark place. Subsequently the absorbance of the solutions was measured at 515 nm against a blank. The percent inhibitory of free radicals was calculated by equation (I):

(I) I% = ((A_b_ – A_s_) / A_b_) × 100

Where I% is the radical scavenge activity, A_b_ is the absorbance of the control sample and A_s_ is the absorbance of test sample. The EC_50_ was calculated from the curve of radical scavenge activity (I%) vs. sample concentration. In this work we used the ascorbic acid as positive control and repeated the test three times.

#### 3.4.2. β-Carotene Linoleic Acid System

The antioxidant activity of extracts was determined by using β-Carotene linoleic acid system according to the method earlier described by Thitilertdecha (26). Briefly, 3 mL of linoleic acid solution (containing 10 mg mL^-1^ in chloroform), 1.5 mL of β-carotene solution (containing 1 mg mL^-1^ in chloroform), and 1.0 mL of Tween 40 solution (containing 300 mg mL^-1^ in chloroform) were completely mixed into a 250 mL flask, then the mixing was stopped at room temperature for 15 minutes. The chloroform was removed; the resulting mixture was topped up to 150 mL using oxygenated deionized water and was shaken to form a β-carotene–linoleic acid emulsion. Aliquots (2.5 mL) of emulsion were pipetted into test tubes containing 0.5 mL of each extracted in methanol (2 mg mL^-1^). Then all tubes were vigorously shaken and incubated at 50 °C in a water bath. After that, the absorbance was measured at zero time (A0) at 490 nm by a spectrophotometer. After 120 minutes, the second absorbance (At) was measured. The blank was a mixture of above solution without β-carotene and used for background subtraction. The antioxidant activity (AA%) of the sample was measured by equation (II) as follows:

(II) AA% = ([ 1 – (S_A0_ - S_At_)] / (C_A0_ - C_At_)) × 100

Where the S_A0_ and C_A0_ were the absorbance values of sample and the control at 0.0 minute, respectively; S_At_ and C_At_ were the absorbance values of sample and control after 120 minutes, respectively. In this section the BHT was used as positive control.

#### 3.4.3. Reducing Power Ability

The reducing power of polar and non-polar extracts from plant was determined spectrophotometrically according to the work of Ahmadi (14) with some modifications. Different concentrations of methanolic extracts (1.0 mL) were added to 2.5 mL of potassium ferrocyanate [K_3_ Fe(CN)_6_] (1%) and 2.5 mL of phosphate buffer (0.2 M, pH = 6.6). The mixture was incubated for 20 minutes at 50 ºC. To stop the reaction, 2.5 mL of trichloroacetic acid (10%) was added and the mixture was then centrifuged at 3000 rpm for 10 minutes. Then 2.0 mL of supernatant of solution was removed and mixed with 2.5 mL of distilled water and 0.5 mL of ferric chloride (0.1%). The absorbance of solution was measured at 700 nm. The ascorbic acid was used as positive control. The increase of absorbance is synonymous of an increase in reducing power.

#### 3.4.4. The Measuring of Total Phenolic Contents 

The Folin–Ciocalteu reagent and gallic acid as standard were used for determination of total phenolic contents of polar and nonpolar extracts in accordance with the Cabral de Oliveira method (27). In two volumetric flasks, 0.1 mL of solutions containing 500 μg of each extract, 46 mL of distilled water and 1.0 mL of Folin–Ciocalteu reagent were added and the flasks were thoroughly shaken. Following 3 minutes each solution reached to 50 mL by 2%w/v of Na_2_CO_3_ solution and incubated for 2 hours at room temperature. Then the absorbance of each mixture was measured at 760 nm. The standard curve was obtained by using the same procedure for standard solutions of gallic acid. The total phenol compounds were expressed as µg gallic acid per mg of extract. The experiments were carried out in triplicate, and Gallic acid equivalent values have been reported as X ± SD.

## 4. Results

### 4.1. Total Phenolic Content

From the 20 g of all parts of plant that subjected to solvent extraction, 7.87 g solid green extract was obtained (39.35%w/w). Correspondingly the extraction yields of resultant solid compounds, into the aqueous and chloroform phases were 73.44% and 26.55%, respectively. One of important constituents of plants is the polyphenolic compounds due to their strong antioxidant activity. This activity is mostly performed by preventing hydroperoxide conversion into reactive oxyradicals, chelating redox-active metal ions and inactivating lipid free radical chains. The amounts of phenolic compounds in polar and nonpolar subfraction of *Mindium laevigatum* were measured by the Folin and Ciocalteu reagent and expressed as gallic acid equivalents. It is well known that the Folin–Ciocalteu reagent is a yellow acidic solution containing phosphomolybdic and phosphotungstic heteropoly acids. Following oxidation of phenolates by this reagent, a blue complex of molybdenum-tungsten was produced that could be detected at 760 nm. The data presented in Table 1 indicates that the amount of total phenol in polar subfraction of extract was higher than nonpolar ones. The high phenolic content in polar subfraction of methanol extract contributes to its increased antioxidant potential in comparison to non-polar subfraction. As seen from the results, there is a strong relationship between the quantities of phenolic compounds with antioxidant activity. The phenolic compounds easily donate the hydrogen atoms to free radicals and stopped the chain reaction of lipid oxidation. This scavenging of radicals is may be due to the presence of phenolic hydroxyl groups.

**Table 1:** Antioxidant Capacities and Total Phenol of *M. laevigatum*

Sample	Test System		
	DPPH EC50 value, µg.mL^-1^	β-Carotene/linoleic acid inhibition, %	Total phenol, µg.gr^-1^
**Polar subfraction**	175 ± 5.2	68.25 ± 0.67	182 ± 4.2
**Non-polar subfraction**	250 ± 7.4	42.3 ± 0.39	158 ± 3.9
**BHT**	-	89.9 ± 0.67	-
**Ascorbic Acid**	50 ± 2.1	-	-

EC_50_ values: The effective concentration at which the antioxidant activity using the DPPH radicals were scavenged by 50% and EC_50_ values were obtained by interpolation from linear regression analysis. Each β-carotene/linoleic acid inhibition and total phenol value is expressed as Mean ± standard deviation (n = 3)

### 4.2. Antioxidant Properties

#### 4.2.1. DPPHº Radical Scavenging Activity

**Figure 1:**
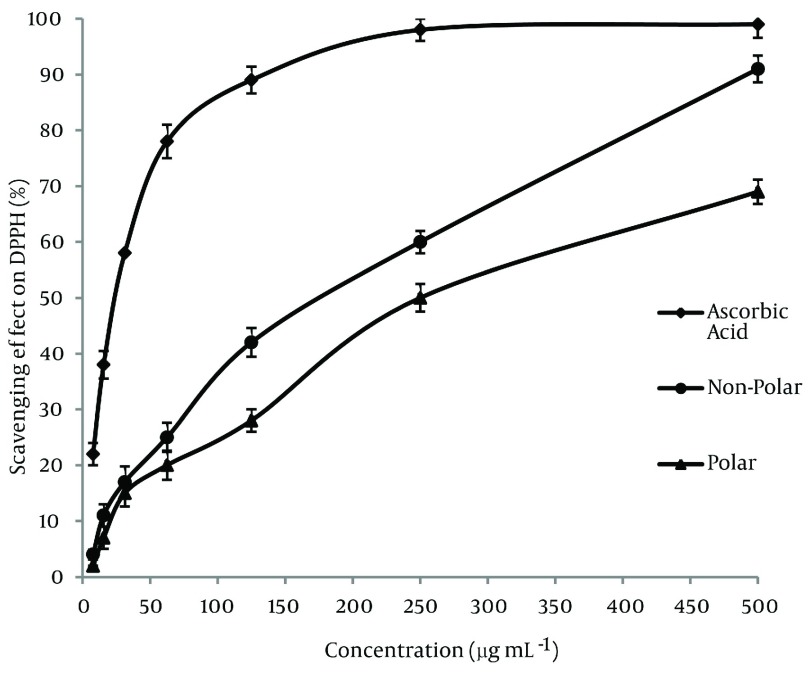
Free Radical Scavenging Properties of the Methanolic Sub-Fractions of *M. laevigatum* (vent.) Rech. F.

The DPPHº radical scavenging assay was used in order to comparing the powerful antioxidant activity of subfraction extracts of *Mindium laevigatum* with ascorbic acid as standard. A freshly prepared DPPHº solution displays a deep purple color (λ max = 515 nm) that gradually vanishes in the presence of a good hydrogen donor, i.e., a potent antioxidant. The DPPHº-scavenging activities (%) of polar and nonpolar subfractions extracts were compared to ascorbic acid and shown in (Figure 1). Moreover in this study the DPPHº scavenging activity of extracts were represented by EC_50_ value (concentration of the antioxidant needed to scavenge 50% of DPPHº present in the solution and lower EC_50_ values reflect better DPPHº radical scavenging activity) and the data placed at Table 1 . As it is observed, the polar sub-fraction of methanolic extract of *Mindium laevigatum* has the highest radical scavenging activity with EC_50_ value of 165 ± 3.1 (µg mL^-1^). The data resulted that the DPPHº scavenging activity of extracts increased in the order of: ascorbic acid > polar sub-fraction > non-polar sub-fraction.

#### 4.2.2. β-Carotene–linoleic Acid Assay

Also, the antioxidant activities of plant extracts were reevaluated by β-Carotene–linoleic acid assay. From the theoretical point of view, the linoleic acid free radicals have a tendency to attack unsaturated β-carotene, while the presence of antioxidants in the solution prevent this extent of β-carotene-bleaching by neutralizing the linoleate free radical and other free radicals formed in the system. This caused the absorbance decrease rapidly in samples without antioxidants, whereas in the presence of an antioxidant the color was retained for a long time. The relative anti-oxidative activities (AA%) of the extracts were calculated from the equation (II). Calculated AA% of the extracts has been presented in Table 1 . Inhibition values of linoleic acid oxidation were estimated as 68.25% and 42.3% in the presence of polar and non-polar sub-fractions of methanolic extracts, respectively. We have known that the basis of β-carotene/linoleic acid bleaching test is the inhibition of lipid peroxidation by donation of a hydrogen atom. Therefore, the compounds which contain hydrogen atoms in the allylic/benzylic positions show better antioxidant activity. This is due to the relatively easy abstraction of atomic hydrogen from these functional groups by peroxy radicals formed under testconditions. High bleaching activity of plant extracts in this test may also be a consequence of the presence of allylic/benzylic containing compounds. According to the results obtained, polar subfraction was found the most active one with an EC_50_ value of 175 ± 5.2 µg mL^-1^. EC_50_ value of ascorbic acid and %AA of the synthetic antioxidant BHT were also determined in parallel experiments. None of the extracts showed activity as strong as the standards.

#### 4.2.3. Reducing Power Assay

**Figure 2:**
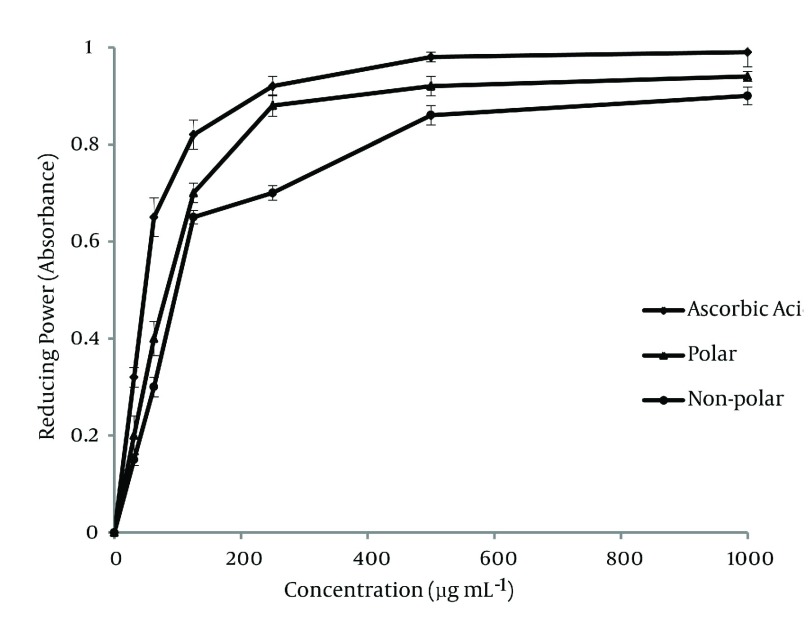
The Reducing Power of Various Concentration of Sub-fraction Extracts

The antioxidant activities of polar and non-polar extracts were investigated for the reductive capabilities by using the potassium ferricyanide reduction method. The resultant data for the various concentrations of the two extracts are shown in Figure 2 . In this experiment, depending on the reducing power of each extract, the yellow color of the solution vanished and changed to various shades of green and blue. The presence of antioxidants in the solution caused the Fe_3_+/ferricyanide complex reduced to the Fe_2_+ form and the color of solution changed to a Perl’s Prussian blue (Fe_4_ [Fe(CN)_6_]_3_). The concentration of Fe_2_+ can be measured at 700 nm. As the ion of Fe_3_+ reduced to Fe_2_+ ion, therefore, this reduction is often used as an indicator of electron donating activity of phenolic compounds. In the reducing power assay, the increasing absorbance at 700 nm indicates an increase in reductive ability. As it is observed, the non-polar subfraction has restored antioxidant activity.

### 4.3. Antibacterial and Antifungal Activities

*Candida albicans* is an opportunistic pathogen responsible for about 60% of both superficial and systemic mycoses (28), and it is capable of reversible transition between the yeast and hyphal form. Also, it is well known that the *Aspergillus niger* is a more frequent agents of pulmonary aspergillusis and otomycosis (29). In addition, the *S. epidermidis, P. aeruginosa, Morganella*, and *C. freundii* are the opportunistic bacteria and *E. feacalis, S. aureus*, and *E. coli* are true human pathogens (30). Therefore, the antibacterial and antifungal activities of polar and non-polar subfractions of the plant under study against these pathogenic microorganisms were investigated by disc diffusion and MIC methods. As can be revealed in Table 2, the polar and non-polar fraction shows a significant activity against the *Staphylococcus aureus*. The results from the measurements of minimal inhibition concentration (MIC) indicate that the non-polar sub-fraction has more antibacterial effect on *Staphylococcus aureus* with a MIC value of 120 µg mL^-1^. Furthermore, we determined a resistance of *E. feacalis, S. epidermidis, P. aeruginosa, Morganella, C. freundii*, and *E. coli* microorganisms against the non-polar sub-fraction of plant. This weak spectrum of antibacterial activity of polar and non-polar subfractions may be due to low or very rare presence of antibacterial constituents. In addition, the synergistic and/or antagonistic effects of presence compounds in the extracts might be have remarkable effects for the activity observed in complex systems. It is well known that the *S. aureus* is one of the important food-borne pathogen and is threat to global public health. It causes poisoning and gastroenteritis and is found in a wide variety of foodstuffs. Cooking pasteurization are suitable methods for eliminating of *S. aureus*, but heat treatment is not desirable for all foods and cross-contamination cannot always be prevented. Elimination and control of the growth of *S. aureus* is important objective for different sectors of the food production industries. Therefore, *Mindium laevigatum* might be able to be used as a natural preservative against *S. aureus* for the food industries. Table 3 represents the effects of extracts on the growth of three different pathogenic fungal strains. As it has been observed, both polar and non-polar subfractions have significant inhibitory effects on the growth of fungi. It is well known that the common chemical groups in the methanolic extract of the plants are the tannins, flavonoids, alkaloids, and heterosides (31). From the investigation by Tsuchiya (32), relating the antifungal effects of flavonoids and their capacity to form complexes with extracellular and soluble proteins and with cell walls, it may be suggested that the presence of flavonoids in the methanolic extract may play a role in the observed antifungal activity.

**Table 2:** Anti-microbial Capacities of *Mindium laevigatum* Species

Pathogenic Bacteria	Polar subfraction	Non-polar subfraction
	MIC a	MIC a
***E. feacalis***	> 800	> 800
***S. epidermidis***	> 800	> 800
***P. aeruginosa***	> 800	> 800
***Morganella***	> 800	> 800
***C. freundii***	> 800	> 800
***E. coli***	> 800	> 800
***S. aureus***	360	120

^a^MIC, Minimal Inhibitory Concentration, µg mL

**Table 3:** Antifungal Capacities of *Mindium laevigatum* Species

Pathogenic fungal	Polar subfraction			Non-polar subfraction		
	MIC b	Disc diffusion a	Ketoconazole	MIC	Disc diffusion c	Ketoconazole
***Aspergillus niger***	400	14 ± 0.3	32 ± 0.4	400	18 ± 0.1.1	32 ± 0.4
***Candida albicans***	100	24 ± 0.9	40 ± 0.8	50	34 ± 1.9	40 ± 0.8
***Aspergillus candidus***	200	20 ± 0.7	28 ± 0.6	100	26 ± 1.2	28 ± 0.6

^a^Values expressed are Mean ± SD of three parallel measurements

^b^MIC, minimal inhibitory concentration (as µg.mL^-1^)

^c^Diameter of inhibition zone including disc diameter of 10 mm

## 5. Discussion

Today there are numerous reports on the evaluation of antifungal, antibacterial and antioxidant of various plant species in literature, but all plants are not suitable as food preservatives because the safety and toxicity of reported plants needs to be addressed. However, a wide spectrum of antioxidant and antifungal activity of polar and nonpolar subfraction of *M. laevigatum* is reported in the present study for the first time. In conclusion, the development of natural antimicrobial and antioxidants will assist to decrease the negative effects (residues, resistance, and environmental pollution) of synthetic antioxidants such as BHT and BHA and synthetic drugs. In this respect, natural compounds may be also effective, selective, biodegradable, and less toxic to the environment. The methanolic extract of *M. laevigatum* had wide spectra of considerable antioxidant and antifungal activities; while their antibacterial effects are relatively low except for *S. aureus*. In view of the present results, it is concluded that these polar and non-polar methanolic extracts can be used as antifungal and antioxidant agents in medicine and the food industry. However, the safety and toxicity of these compounds will need to be addressed; also the active compounds will need to be recognized.
